# 1109. Case Study. Successful Burn Wound Management with Bioresorbable Antimicrobial Matrix over Split-thickness Skin Graft

**DOI:** 10.1093/jbcr/irag033.213

**Published:** 2026-04-06

**Authors:** Carmen E Flores

**Affiliations:** Leon S. Peters Burn Center, Las Vegas, NV

## Abstract

**Patient Presentation (age range, injury details, relevant history):**

This case involves a 21-year-old male with 70% TBSA thermal burns to the face, neck, and extremities following a motor vehicle accident with explosion. Over the next five months, his course was complicated by Stage IV pressure ulcers of the head, sacrum, and feet, recurrent infections, autograft failures, and exposed avascular structures. He was then transferred to our facility post burn month 5, at which point only ~10% of his burns had closed.

**Clinical Challenges:**

Infection is an independent predictor of graft loss, and up to 75% of burn mortality is attributable to infection. Prior evidence shows that a fully synthetic, bioresorbable antimicrobial matrix containing metallic and ionic silver can reduce surgical site infection risk in complex patients. This report describes its use in a severely burned patient with a complicated pre-transfer course, recurrent infection, and delayed closure.

**Management Approach:**

Remaining open burn and donor site wounds on the posterior torso were excised along with the sacral ulcer. Interventions included epidermal autograft spray, a skin substitute, and the antimicrobial matrix. The torso was dressed with methylene blue/gentian violet foam and absorptive dressings, while negative pressure wound therapy was applied to the sacral wound. Concurrently, sheet autografts were placed to the left hand and forearm, right buttock, and neck. The antimicrobial matrix was immediately applied over autografts, then covered with silver nitrate wet-to-moist dressings. Additional applications of the matrix and autografts were required due to healing complications.

**Outcomes:**

The patient required two return trips to the operating room to close his pre-existing posterior torso donor site wounds, which were infected at transfer. Following repeat matrix application and adjunctive measures, infection resolved, and the wound bed was optimized for autografting. At 7 weeks post-index operation, autografting was performed for areas complicated by shearing and infection related to difficulty with offloading the torso. At 9 weeks, the remaining open donor wounds were successfully grafted. The patient was discharged to rehabilitation three months after the first operation at our facility.

**Lessons Learned:**

Combined with multimodal wound care strategies, the bioresorbable antimicrobial matrix provided effective empiric coverage of autografts in previously infected, hypergranular beds, supporting graft take. On donor sites of the posterior torso, it also facilitated wound bed preparation, reduced graft requirements, and improved closure outcomes.

**Applicability to Practice:**

This case demonstrates that the bioresorbable matrix can complement or replace standard antimicrobial approaches, reduce infection risk, and support autografting readiness in complex burn management.

